# Topical Dinoprostone vs. Foley’s Catheter: A Systematic Review and Meta-Analysis of Cervical Ripening Approaches

**DOI:** 10.3390/healthcare13090983

**Published:** 2025-04-24

**Authors:** Amal Yaseen Zaman, Howaida Amin Hassan, Nageshwar Venkatesh Reddy, Farzana Begum, Samar Ahmed Mahmoud, Hayat Alghamdi, Naglaa Kamel AbdAllah Hussein, Mariam Yousif Elhussain, Soad Mohamed Alnassry, Magda Mubarak Merghani, Manal Elzein Musa, Hanan Mohammed Mohammed, Hammad Ali Fadlalmola

**Affiliations:** 1 Department of Obstetrics and Gynecology, College of Medicine, Taibah University, Madinah 42353, Saudi Arabia; amalyzaman@hotmail.com; 2 Department of Obstetrics and Gynecological Nursing, Alriyada College for Health Sciences, Jeddah 23332, Saudi Arabia; dr_howaidaamin@yahoo.com; 3 Department of Mental Health and Psychiatric Nursing, Faculty of Nursing, Al Baha University, Al Baha 65799, Saudi Arabia; nreddy@bu.edu.sa; 4 College of Nursing, Taif University, Taif 21944, Saudi Arabia; farzana.skgm@gmail.com; 5 Department of Community Health Nursing, Faculty of Nursing, Al-Baha University, Al-Baha 1988, Saudi Arabia; samar@bu.edu.sa (S.A.M.); h.alghamdi@bu.edu.sa (H.A.); naglaa.kamel@nur.dmu.edu.eg (N.K.A.H.); hanan.tab2023@yahoo.com (H.M.M.); 6 Nursing College, University of Hafr AL Batin, Hafr Al Batin 257689, Saudi Arabia; myomer@uhb.edu.sa (M.Y.E.); mmmahmed@uhb.edu.sa (M.M.M.); 7 Department of Nursing, College of Nursing and Health Sciences, Jazan University, Jazan 45142, Saudi Arabia; hibasoaad10@gmail.com; 8 Department of Obstetrics & Gynecological Nursing, Faculty of Applied Medical Sciences, Buraydah College, Buraydah 51418, Saudi Arabia; manalelzein83@gmail.com; 9 Department of Community and Public Health, Nursing College, Taibah University, Madinah 42377, Saudi Arabia

**Keywords:** cervical ripening, dinoprostone, Foley’s catheter, meta-analysis, systematic review

## Abstract

**Background and aim**: Labor induction is increasing in obstetric practice. In women with an unfavorable cervix, cervical ripening is required for successful induction. We conducted this review to compare the effectiveness and tolerance of two interventions used for cervical ripening, topical prostaglandin E2 (dinoprostone) and transcervical Foley’s catheter. **Methods**: We systematically searched four biomedical databases on 15 November 2024, for relevant studies. The studies’ eligibility was determined after screening their titles, abstracts, and full texts. We extracted relevant data from the studies included. RevMan software V5.4 was used to conduct the random-effect meta-analysis. Misoprostol was excluded from this review due to variability in dosing protocols and inconsistent reporting across studies. A review protocol was registered with the International Prospective Register of Systematic Reviews (PROSPERO), registration number: CRD420251026183. **Results**: This review included 41 studies that in total enrolled 12,877 women (6722 for Foley’s catheter and 6155 for dinoprostone). The efficacy of the two interventions was comparable as evidenced by the similarity in the induction to delivery time, Bishop score change, the rate of vaginal delivery within 24 h, and the risk of induction failure. Foley’s catheter was linked with lower risks of caesarian delivery (RR = 0.84, *p* = 0.006), uterine hyperstimulation (RR = 0.39, *p* < 0.001), postpartum hemorrhage (RR = 0.76, *p* = 0.03), and a 1-min Apgar score < 7 (RR = 0.75, *p* = 0.02). However, it was associated with an extra need for oxytocin augmentation (RR = 1.18, *p* < 0.001). The risks of instrumental delivery, intrapartum pyrexia, postpartum infection, meconium passage, umbilical cord arterial pH < 7.1, a 5-min Apgar score < 7, and neonatal intensive care requirement were comparable for the two interventions. **Conclusions**: In comparison with dinoprostone, Foley’s catheter is equally effective and well tolerated. We recommend Foley’s catheter use for women with a previous caesarian delivery and in low-resource settings.

## 1. Introduction

The target of obstetric care is a conception that ends with delivering a healthy baby while maintaining the well-being of the mother. Sometimes, due to obstetric or medical causes, the risks of continuing the conception–for the mother or the fetus–outweigh the risks of immediate delivery. At these times, induction of labor (IOL) is indicated [1]. Labor induction encompasses iatrogenic stimulation of regular uterine contraction prior to spontaneous labor onset, aiming at vaginal delivery within 24 to 48 h [2]. Besides elective timing of birth, the most frequently encountered indications for IOL include postmaturity, gestational diabetes and hypertensive disorders, oligo- or polyhydramnios, as well as other pregnancy complications [3,4]. Globally, IOL is the most common and important obstetric intervention, applied in 20% to 30% of pregnancies [5,6]. The rate of IOL has tripled since 1990, and it is expected to further increase in the future [7,8]. Thus, more data on the most effective and safe approach for IOL is significantly required.

As pregnancy evolves towards term, physiological changes occur in the cervix resulting in a less resistant favorable (ripe) cervix that allows for uterine contents evacuation [9]. The outcome of IOL is to a great extent dependent on cervical status in terms of dilatation, position, effacement, and consistency [10]. In the presence of a ripe cervix, IOL is conducted via amniotomy and oxytocin infusion. However, in women with an unripe cervix, these methods alone would result in a prolonged induction–delivery interval, induction failure, and increased childbirth interventions (including caesarian delivery) [11]. In such cases, preparing the cervix for vaginal delivery by inducing effacement and dilatation, a process known as cervical ripening, is essential prior to amniotomy or oxytocin administration [11,12].

Artificial cervical ripening can be achieved via pharmacological or mechanical methods. Pharmacological methods include oral or vaginal administration of exogenous prostaglandins (E1 or E2), estrogen or progesterone antagonists [13]. The most common pharmacological agent in use for cervical ripening is the prostaglandin E2 (PGE2) otherwise known as dinoprostone [14]. In most body tissues, PGE2 presents naturally in low concentrations and acts as a local hormone [15]. Dinoprostone exerts its effects by producing procollagenase, a collagenase precursor. This reduces cervical collagen and increases hyaluronic acid resulting in cervical softening that eases effacement and dilatation [16]. Topical dinoprostone is available in the forms of gel, tablet, suppository, and controlled-release insert. On the other hand, mechanical methods for cervical ripening include the use of hygroscopic laminaria dilators and balloon catheters with or without an extra-amniotic infusion of saline. The most frequently used mechanical method is the transcervical single balloon catheter (Foley’s catheter) [17]. The catheter is inserted beyond the internal os and its balloon is then inflated to mechanically stretch the cervical tissue and fetal membranes. This mechanical stretching results in the release of endogenous prostaglandins and subsequent cervical ripening [18,19].

Several studies were conducted to compare methods used for cervical ripening. Especially, studies compared the frequently used pharmacological and mechanical methods; topical PGE2 and transcervical Foley’s catheter. However, the studies’ findings were inconsistent and consensus is yet to be reached on the optimal cervical ripening approach. While making such a comparison, both efficacy and safety profiles as well as maternal and fetal outcomes should be taken into consideration. In this view, we conducted this systematic review and meta-analysis aiming to compare the effectiveness and tolerance of topical dinoprostone and transcervical Foley’s catheter for cervical ripening in women with unripe cervix indicated for IOL.

## 2. Methods

In this review article we defined explicit inclusion and exclusion criteria prior to study selection and applied them consistently during screening. All methodological procedures followed PRISMA and the Cochrane Handbook for Systematic Reviews of Interventions (version 6.3) [20,21]. A review protocol was registered with the International Prospective Register of Systematic Reviews (PROSPERO), registration number: CRD420251026183.

### 2.1. Literature Search and Selection of Studies

On the 15 November 2024, we searched four biomedical databases in a systematic manner for studies eligible for this review. We used the following terms in our search: (“prostaglandins E2” OR “prostaglandin E2” OR “PGE-2” OR “PGE2” OR dinoprostone) AND (“Foley catheter” OR “Foley’s catheter” OR “Foley’s” OR “Foley Balloon Catheter”). We gathered all search results and removed the duplicates in preparation for screening. The studies’ titles and abstracts were initially screened to select studies eligible for full-text screening, which was conducted thereafter.

We selected studies that compared topical dinoprostone with transcervical Foley’s catheter for cervical ripening in women with an unripe cervix planned for IOL. All preparations of vaginal/intracervical dinoprostone were studied. Similarly, all sizes of Foley’s catheter and degrees of balloon inflation were explored. Studies available only as an abstract, theses, books, animal studies, and non-English/Arabic studies were excluded. Finally, we reviewed the reference lists of the included reports in search of more eligible studies.

Fourteen studies were excluded after full-text screening due to reasons such as insufficient outcome data, duplicate reporting, non-comparative designs, or failure to meet the predefined inclusion criteria.

### 2.2. Quality Assessment

The risk of bias evaluation tool provided by the Cochrane Collaboration was used to judge the quality of the eligible controlled clinical trials. Their tool evaluates the risks of bias in patient selection, procedures performance, outcomes detection, participants’ attrition, and trial reporting as well as other potential sources of bias [20]. In regard to cohort and case-control studies, we evaluated their quality using tools provided by the National Institute of Health. Via answering questions about the studies’ methods and reports, a total score for each study is calculated and a corresponding quality judgment is given. The studies’ quality was described as “good”, “fair”, or “poor” [22].

### 2.3. Data Extraction

To describe the studies included in this review, we extracted data on the studies’ design, site, sample size, patients’ eligibility criteria, and the Foley’s catheter size and the extent of its balloon inflation, in addition to the used dinoprostone preparation and its dose. Moreover, we extracted further data to describe women enrolled in each study. This data included women’s age, body mass index (BMI), parity, gestational age, baseline Bishop score, and the indication for IOL. Bishop’s score is the gold standard in cervical evaluation as it estimates cervical readiness for IOL. The score evaluates the position, dilatation, effacement, and consistency of the cervix besides the presenting part station [23]. A Bishop score of less than six indicates an unfavorable cervix and a high probability of induction failure unless cervical ripening is performed [24].

Outcomes explored in this review included the change in Bishop score, time interval from induction to delivery, vaginal delivery within 24 h, induction failure, oxytocin augmentation, caesarian delivery, instrumental vaginal delivery, uterine hyperstimulation or tachystole, intrapartum pyrexia, postpartum infections, postpartum hemorrhage, meconium passage, umbilical cord arterial pH < 7.1, Apgar score < 7 at one and five minutes, and neonatal intensive care unit (NICU) admission.

### 2.4. Quantitative Synthesis

We conducted our meta-analysis in the inverse variance method using RevMan software V5.4. Our analyses used the mean difference (MD) as the effect estimate in pooling continuous outcomes, whereas the risk ratio (RR) was the effect estimate for categorical outcomes. For all analyses, the effect estimate was provided beside its 95% confidence interval (CI) and the *p*-value. The *p*-value is used to determine significance which was considered from a *p*-value < 0.05. The included studies encompassed a large degree of heterogeneity in their methodology. Thus, we conducted our meta-analysis using the random-effect model as suggested by Cochrane’s handbook [20]. The studies’ findings heterogeneity was evaluated via the Chi-squared and the I-squared (I2) statistics, where a Chi-square statistic *p*-value < 0.1 and an I2 statistic ≥ 50 implied a considerable level of heterogeneity [25,26].

## 3. Results

### 3.1. Literature Search and Selection of Studies

**Figure 1:**
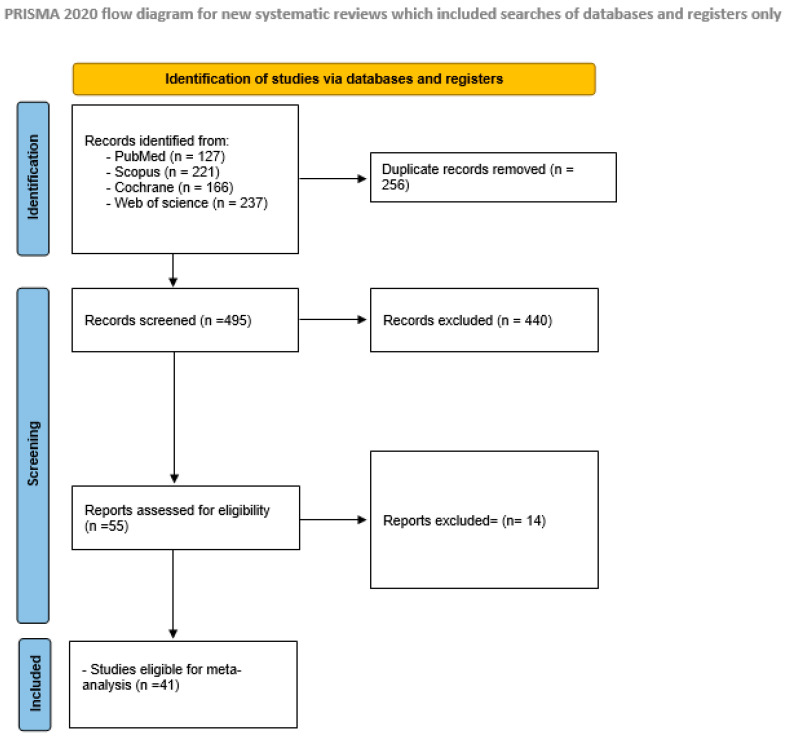
PRISMA flow diagram.

The applied systematic search entirely retrieved 751 results. Following duplicate removal, 495 articles underwent title and abstract screening. Of those, 55 studies were eligible for full-text screening. Eventually, we included 41 studies in the qualitative and quantitative production of this review [27,28,29,30,31,32,33,34,35,36,37,38,39,40,41,42,43,44,45,46,47,48,49,50,51,52,53,54,55,56,57,58,59,60,61,62,63,64,65,66,67]. As shown in Figure 1.

### 3.2. Studies’ Description

We included 29 controlled clinical trials [27,28,29,30,32,34,40,42,45,46,47,48,49,50,51,52,53,54,55,56,57,58,60,61,62,63,64,65,66], 11 cohort studies [31,33,35,36,37,38,39,41,44,59,67], and one case-control study [43]. The selected studies represented the populations of 17 countries; the United States, Australia, Canada, China, France, the United Kingdom, Poland, Spain, Sweden, Italy, Denmark, the Netherlands, Jordan, Iran, India, Nigeria, and Israel. We conducted this review based on data from 12,877 women enrolled in the included studies. Of those, 6722 women underwent cervical ripening with Foley’s catheter whereas 6155 underwent ripening with topical dinoprostone. The Foley’s catheter sizes ranged from 14 to 24 French, and the balloon was inflated with 30 to 150 mL of saline or distilled water. As shown in Table 1.

The average age of enrolled women ranged from 21 to 35 years, and the majority of studies included normal to overweight women according to their average BMI. In most of the included studies, the greatest proportion of women were primigravidae, and the mean gestational age was between 38 and 41 weeks. Generally, postmaturity was the most common indication for IOL, followed by gestational hypertensive disorders, oligo- or polyhydramnios, intrauterine growth restriction, and gestational diabetes. Mean patients’ baseline Bishop score ranged from 1.5 to 4.5. As shown in Table 2.

### 3.3. Quality Assessment

All the controlled trials involved in this review had a low risk of patient selection bias except three trials that were not randomized [28,50,60]. In some trials, the method of concealing patients’ allocation was not described resulting in an unclear risk of bias judgment [29,34,40,42,49,52,55,56,57,66]. In addition, all the included controlled trials had low risks of participants’ attrition bias and selective outcomes reporting. However, none of the trials applied blinding in their methodology, a point that might add a risk of performance and detection biases. Another potential source of bias is the absence of a published protocol in many studies [28,29,30,32,34,40,46,49,50,51,52,54,55,56,57,58,60,61,62,63,64,65,66] (Figures S1 and S2). All the included cohort studies and the case-control study were judged to be of high quality regarding their methods and reports (Tables S1 and S2).

### 3.4. Efficacy and Safety Outcomes

#### 3.4.1. Caesarian Delivery

**Figure 2:**
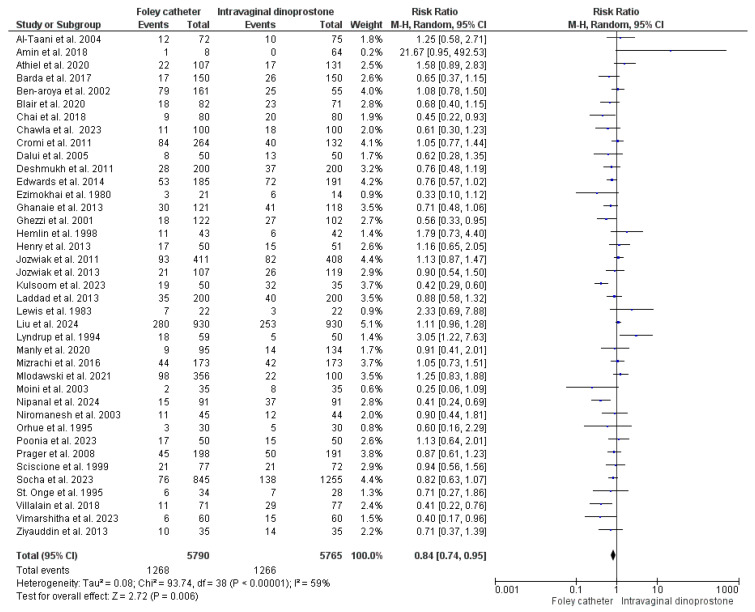
Forest plot of the analysis; Caesarian delivery [27,28,29,31,32,33,34,35,36,37,38,39,40,41,42,43,45,46,47,48,49,50,51,52,53,54,55,56,57,58,59,60,61,62,63,64,65,66,67].

An analysis of caesarian delivery risk was conducted on data from 11,555 women reported in 39 studies (5790 women underwent ripening with Foley’s catheter and 5765 with dinoprostone) [27,28,29,31,32,33,34,35,36,37,38,39,40,41,42,43,45,46,47,48,49,50,51,52,53,54,55,56,57,58,59,60,61,62,63,64,65,66,67]. This analysis showed the superiority of Foley’s catheter over topical dinoprostone (RR = 0.84, 95%CI: [0.74, 0.95], *p* = 0.006), but the studies’ findings were heterogeneous (*p* < 0.001, I^2^ = 59%). As shown in Figure 2.

#### 3.4.2. Instrumental Vaginal Delivery

Twenty-two studies with 5180 women enrolled (2810 for Foley’s catheter cervical ripening and 2370 for dinoprostone ripening) were involved in this meta-analysis [28,29,34,35,38,43,47,48,49,50,51,52,53,54,55,56,59,62,63,64,65,67]. Analysis results revealed a homogenously insignificant variation between the two interventions in the risk of assisted vaginal delivery (RR = 0.89, 95%CI: [0.75, 1.05], *p* = 0.17), (*p* = 0.47, I^2^ = 0%). As shown in Figure S3.

#### 3.4.3. Induction to Delivery Interval (Hours)

This outcome was reported in 30 studies, with 7822 women enrolled (3983 for Foley’s catheter and 3884 for dinoprostone) [27,28,29,31,32,34,36,37,38,39,41,43,45,46,47,48,49,51,52,53,54,56,57,58,61,62,63,64,65,67]. Our analysis concluded an insignificant difference between Foley’s catheter and dinoprostone in the time required for delivery (MD = −0.67, 95%CI: [−3.10, 1.77], *p* = 0.59). The studies’ findings on this outcome were noticeably heterogeneous (*p* < 0.001, I^2^ = 99%). As shown in Figure S4.

#### 3.4.4. Vaginal Delivery Within 24 h

We conducted this meta-analysis on data provided by nine studies that reported 4568 women (2619 underwent ripening with Foley’s catheter and 1949 with dinoprostone) [27,41,43,44,45,47,50,51,65]. According to this analysis, the two interventions were similarly effective (RR = 1.06, 95%CI: [0.85, 1.32], *p* = 0.6), but a significant variation across the studies’ findings was detected (*p* < 0.001, I^2^ = 88%). As shown in Figure S5.

#### 3.4.5. Oxytocin Augmentation

**Figure 3:**
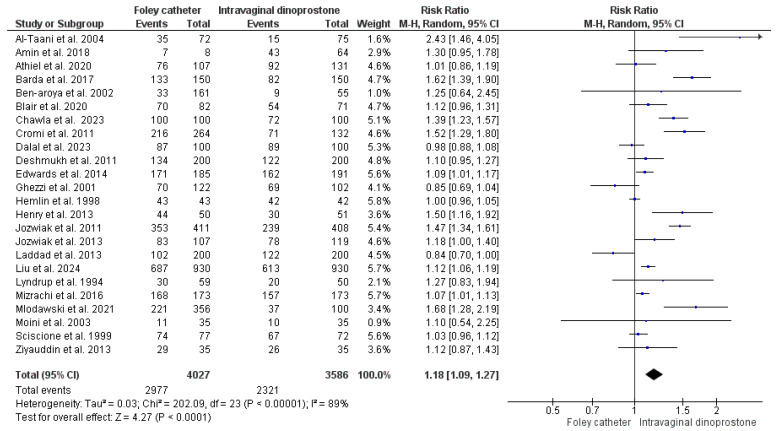
Forest plot of the analysis; Oxytocin augmentation [27,29,30,35,36,37,39,42,43,45,47,48,49,50,51,52,53,57,59,60,61,62,65].

Twenty-four studies reported data on this outcome from a total of 7613 women (4027 were ripened with Foley’s catheter and 3586 with dinoprostone) [27,29,30,35,36,37,39,42,43,45,47,48,49,50,51,52,53,56,57,59,60,61,62,65]. Women who underwent cervical ripening with Foley’s catheter were more likely to require labor augmentation with oxytocin when compared to those ripened with dinoprostone (RR = 1.18, 95%CI: [1.09, 1.27], *p* < 0.001). A considerable heterogeneity was noticed across the included studies’ findings (*p* < 0.001, I^2^ = 89%). As shown in Figure 3.

#### 3.4.6. Induction Failure

Analysis of this outcome was based on data provided by 23 studies reporting 7361 women (3977 for Foley’s catheter and 3384 for dinoprostone) [27,28,30,32,34,36,39,41,43,44,45,46,48,50,51,52,53,57,58,63,65,66,67]. Our comparison homogenously revealed a similar risk of induction failure by the two interventions (RR = 0.87, 95%CI: [0.70, 1.08], *p* = 0.21), (*p* = 0.26, I^2^ = 15%). As shown in Figure S6.

#### 3.4.7. Bishop Score Change

The two interventions similarly resulted in a Bishop score improvement (MD = 0.16, 95%CI: [−0.50, 0.82], *p* = 0.63). This analysis included 14 studies that enrolled 2477 women (1347 for Foley’s catheter and 1130 for dinoprostone) [30,31,32,40,49,51,52,55,56,57,58,60,61,64]. The findings of these studies showed a considerable level of heterogeneity (*p* < 0.001, I^2^ = 95%). As shown in Figure S7.

#### 3.4.8. Uterine Hyperstimulation/Tachystole

**Figure 4:**
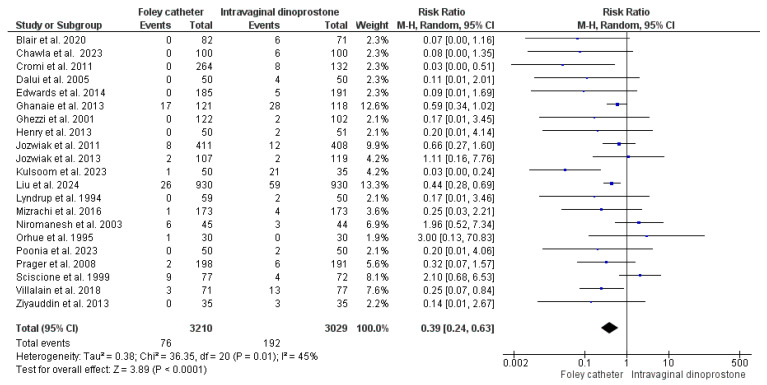
Forest plot of the analysis; Uterine hyperstimulation or tachystole [27,29,31,32,37,41,43,45,46,47,48,50,51,53,54,55,58,60,61,63,65].

This analysis significantly and homogenously revealed a lower risk of uterine hyperstimulation/tachystole with Foley’s catheter use for cervical ripening (RR = 0.39, 95%CI: [0.24, 0.63], *p* < 0.001), (*p* = 0.01, I^2^ = 45%). Data for this analysis were retrieved from 21 studies that reported 6239 women (3210 for Foley’s catheter and 3029 for dinoprostone) [27,29,31,32,37,41,43,45,46,47,48,50,51,53,54,55,58,60,61,63,65]. As shown in Figure 4.

#### 3.4.9. Intrapartum Pyrexia

We conducted this analysis on data provided by 14 studies that involved 5219 women (2589 for Foley’s catheter and 2630 for dinoprostone) [27,32,36,37,38,41,43,46,47,48,53,54,56,60]. The risk of intrapartum pyrexia did not vary significantly between the two groups (RR = 0.89, 95%CI: [0.68, 1.15], *p* = 0.36), a finding consistently observed across the included studies (*p* = 0.28, I^2^ = 16%). As shown in Figure S8.

#### 3.4.10. Postpartum Infection

Analysis of the postpartum infection risk was conducted on the findings of eight studies including 3898 women (1934 were ripened with Foley’s catheter and 1964 with dinoprostone) [27,32,36,43,46,48,50,53]. The analysis showed a homogenously inconsiderable difference between Foley’s catheter ripening and dinoprostone ripening in the risk of postpartum infection (RR = 1.43, 95%CI: [0.93, 2.18], *p* = 0.10), (*p* = 0.39, I^2^ = 5%). As shown in Figure S9.

#### 3.4.11. Postpartum Hemorrhage

**Figure 5:**
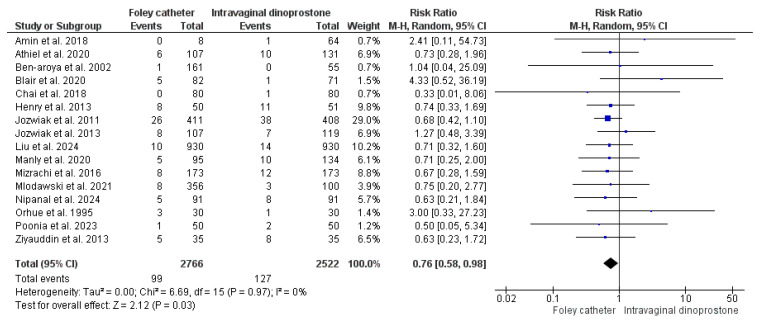
Forest plot of the analysis; Postpartum hemorrhage [27,28,32,35,36,37,38,39,40,43,47,48,50,53,59,63].

Our analysis homogenously revealed a lower risk of postpartum hemorrhage with Foley’s catheter cervical ripening when compared to dinoprostone ripening (RR = 0.76, 95%CI: [0.58, 0.98], *p* = 0.03), (*p* = 0.97, I^2^ = 0%). We retrieved data for this analysis from 16 studies that included 5288 women (2766 were ripened with Foley’s catheter and 2522 with dinoprostone) [27,28,32,35,36,37,38,39,40,43,47,48,50,53,59,63]. As shown in Figure 5.

#### 3.4.12. Meconium Passage

The analysis of meconium passage risk included 16 studies enrolling 4721 women (2553 for Foley’s catheter and 2168 for dinoprostone) [32,35,37,40,43,45,46,48,49,52,53,54,56,57,59,60]. A homogeneously inconsiderable difference in the risk of meconium passage was detected between the two interventions (RR = 1.15, 95%CI: [0.94, 1.41], *p* = 0.17), (*p* = 0.48, I^2^ = 0%). As shown in Figure S10.

#### 3.4.13. Umbilical Cord Arterial pH < 7.1

Twelve studies were included in the analysis of fetal acidosis risk, with 5108 women enrolled (2733 for Foley’s catheter and 2375 for dinoprostone) [27,35,36,41,42,43,45,47,48,51,53,65]. The risk of having an umbilical cord arterial pH < 7.1 was homogenously similar in the two groups (RR = 0.73, 95%CI: [0.50, 1.07], *p* = 0.11), (*p* = 0.74, I^2^ = 0%). As shown in Figure S11.

#### 3.4.14. Apgar Score < 7

**Figure 6:**
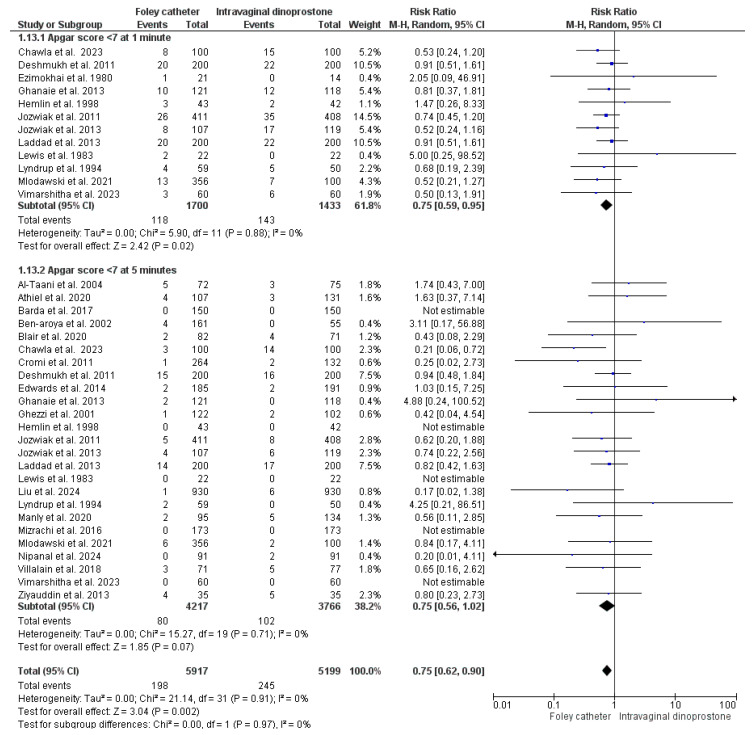
Forest plot of the analysis; Apgar Score < 7 [28,29,34,35,36,37,38,41,42,43,45,46,48,49,51,52,53,56,59,60,62,65,66,67].

The incidence of a 1-min Apgar score < 7 was reported in 12 studies including 3133 women (1700 for Foley’s catheter and 1433 for dinoprostone) [29,34,35,46,48,49,52,53,62,65,66,67]. Our analysis homogenously revealed the superiority of Foley’s catheter (RR = 0.75, 95%CI: [0.59, 0.95], *p* = 0.02), (*p* = 0.88, I^2^ = 0%). The 5-min score was reported in 25 studies that included 7983 women (4217 for Foley’s catheter and 3766 for dinoprostone) [27,28,29,34,35,36,37,38,41,42,43,45,46,48,49,50,51,52,53,56,59,60,62,65,66]. The risk of a 5-min Apgar score < 7 did not differ significantly between the two groups (RR = 0.75, 95%CI: [0.56, 1.02], *p* = 0.07), a finding observed homogeneously across the involved studies (*p* = 0.71, I^2^ = 0%). As shown in Figure 6.

#### 3.4.15. NICU Admission

The risk of NICU admission was homogeneously similar in the two intervention groups (RR = 0.88, 95%CI: [0.73, 1.05], *p* = 0.15), (*p* = 0.96, I^2^ = 0%). This analysis was conducted on data provided by 16 studies that reported 4409 women (2253 for Foley’s catheter and 2156 for dinoprostone) [29,32,34,36,37,41,43,45,46,47,48,49,51,52,53,56]. As shown in Figure S12.

## 4. Discussion

The past years have witnessed a rise in the rate of labor induction, and it is expected to rise further in the coming years [7,8]. Thus, a need to identify optimal approaches for IOL has emerged. The presence of an unfavorable cervix is one of the two main challenges of labor induction [68]. When selecting the best agent for cervical ripening, both maternal and fetal well-being should be taken into consideration. The ideal comparison of cervical ripening interventions equally accounts for efficacy and safety or tolerance. In this view, we conducted this review based on data provided by 41 studies that compared topical dinoprostone with transcervical Foley’s catheter in women with an unripe cervix and an indication for IOL. Ever since the publication of the previous meta-analysis by Zhu et al. in 2018, several comparative studies have been published [69]. Of 41 studies included in this review, only eight were included by Zhu et al. [69]. Unlike the previous meta-analysis, we did not limit this review to randomized controlled trials (RCTs) and primigravidae [69]. Another meta-analysis published by Wang et al. in 2015 was limited to RCTs and the controlled-release preparation of dinoprostone. Wang et al. included only six studies in their meta-analysis [70]. Given that, the present review constitutes a more comprehensive update on the comparison of topical dinoprostone versus Foley’s catheter.

Caesarian delivery is among the most common concerning adverse consequences of labor induction [71,72]. It is performed emergently to save the life of the mother or the fetus. However, the procedure is associated with both short- and long-term adverse consequences for mothers and their offspring. For mothers, caesarian delivery is associated with a prolonged recovery time and complicated subsequent pregnancies (including scar pregnancy, placentation abnormalities, and uterine rupture) [73,74]. Early neonatal consequences include transient tachypnea of the newborn and pulmonary hypertension, whereas the long-term ones include atopy and increased susceptibility to asthma and obesity [75]. Therefore, obstetric care is globally directed to achieve normal vaginal delivery. Contrary to Zhu et al.’s and Wang et al.’s findings, our meta-analysis revealed a lower risk of caesarian delivery with Foley’s catheter ripening. Based on data of 11,555 women, Foley’s catheter reduced the risk of caesarian delivery by 16% when compared to dinoprostone (*p* = 0.006). Zhu et al.’s and Wang et al.’s analysis revealed a similar risk of caesarian delivery with the two interventions [69,70]. Following IOL, caesarian delivery is commonly derived by fetal distress or induction failure [27]. Our analysis did not detect a significant variation between the two interventions in regard to induction failure. This finding is consistent with a meta-analysis that compared various prostaglandins to Foley’s catheter [76]. The risks of meconium passage and fetal acidosis did not vary either between the two interventions. However, Apgar’s score at one minute favored Foley’s catheter ripening. Cervical ripening with Foley’s catheter reduced the risk of a 1-min Apgar score < 7 by 25% when compared to dinoprostone (*p* = 0.02). Wang et al. reported similar findings in regard to meconium passage and fetal acidosis [70]. However, Wang et al.’s analysis of a 1-min Apgar score showed a comparable risk with dinoprostone and Foley’s catheter [70]. A comparison of fetal distress risk using cardiotocography (CTG) findings was not possible because of limited data. Subsequent neonatal outcomes (5-min Apgar score and NICU admission risk) were comparable in the two assessed groups, a finding consistent with Zhu et al. [69].

After the first hour of dinoprostone administration, cervical ripening begins and uterine contractions are noticed [77]. In contrast, Foley’s catheter ripens the cervix with minimal uterine muscle activity [51]. Our analysis detected a less frequent administration of oxytocin for labor augmentation with dinoprostone when compared to Foley’s catheter (RR = 1.18, *p* ≤ 0.001), a finding consistent with Wang et al. [70]. The reduced need for oxytocin administration with dinoprostone comes with the benefits of a reduced need for continuous fetal monitoring and delivery room stay. Nevertheless, the uterine stimulatory effect of dinoprostone is reflected in the risk of uterine tachystole or hyperstimulation. The present meta-analysis detected an increased risk of uterine tachystole or hyperstimulation by 61% when dinoprostone is used for cervical ripening rather than Foley’s catheter (*p* < 0.001). This could also support the hypothesis of fetal distress as a key driver for the increased caesarian delivery risk with dinoprostone. Moreover, this might increase the risk of uterine rupture, especially in women with a previous caesarian delivery [78]. The risk of uterine rupture was not explored in this review, though, due to a scarcity of data. In contrast to our findings, Zhu et al. did not detect a considerable difference between Foley’s catheter and dinoprostone in the risk of uterine hyperstimulation [69]. However, Wang et al.’s findings are consistent with ours [70]. Uterine hyperstimulation might result in a subsequent atony that leads to postpartum hemorrhage. This might explain the 24% increased risk of postpartum hemorrhage among women who underwent cervical ripening with dinoprostone as detected in our analysis (*p* = 0.03). This finding is not consistent with Zhu et al. either [69].

The time interval between initiation of induction and delivery is a key determinant of patients’ satisfaction [79]. In addition, a prolonged duration is associated with greater risks of chorioamnionitis and postpartum pyrexia [80,81]. Our study identified comparable results with dinoprostone and Foley’s catheter in regard to the time interval between initiation of induction and delivery, vaginal delivery within 24 h, Bishop score change, intrapartum pyrexia, and postpartum infection. These findings are consistent with Zhu et al. and Wang et al. [69,70]. However, Wang et al. detected the superiority of dinoprostone in reducing the time interval for delivery and improving the Bishop score [70].

Dinoprostone gel is expensive and requires refrigeration for its storage. Although dinoprostone-mediated cervical ripening has been linked to uterine hyperstimulation and other related consequences, the controlled-release insert preparation is superior as it can easily be removed. After removal, the uterine stimulatory effect is immediately eliminated as the half-life of dinoprostone is short (one to three minutes) [15]. As a prostaglandin, dinoprostone use is contraindicated in women with asthma or an allergy to prostaglandins [29]. On the other hand, Foley’s catheter is cheap and widely available, which makes it ideal for use in low-resource settings. While the minimized uterine stimulation reduces pain associated with Foley’s catheter-mediated ripening, its insertion is more complicated and uncomfortable [35,82]. Despite the low cost of Foley’s catheter, the associated oxytocin need comes with its costs of manpower and delivery room stay. Due to the scarcity of data, we could not conduct a cost-effectiveness analysis to confirm this hypothesis.

This review is powered by the large number of women included, which increases the reliability of our results. Women included in this review represented the populations of 17 countries, reflecting different ethnicities and genetic makeups. Moreover, different preparations of dinoprostone and sizes of Foley’s catheter were explored. The majority of studies involved in this review are RCTs which provide the highest reliable level of evidence, followed by cohort studies that are the best observational study design. The high quality of the included studies added to the strength of this review. Yet, due to the nature of the applied interventions, all the studies’ patients and personnel were not blinded to the interventions. This is suggested to add a risk of bias in the performance as well as bias in the detection of subjective outcomes. Importantly, outcomes explored in this review are yet less likely to be considerably influenced by the open-label design. The included studies are heterogenous in regard to study design, participants’ eligibility criteria, dinoprostone preparation and dose, Foleys’ catheter size and balloon inflation, involvement of extra-amniotic saline infusion, repetition and duration of the interventions, labor management decisions and guidelines, and definitions of the outcomes. Due to the scarcity of the available data, we could not account for these factors in our analysis. Nevertheless, we conducted this meta-analysis using the random-effect model to account for heterogeneity as suggested in Cochrane’s Handbook [20]. The findings of this review can guide obstetricians in their case-specific selection of the most appropriate cervical ripening intervention. We observed a moderate to high level of heterogeneity in some outcomes, likely due to differences in study design, participant characteristics, and intervention protocols. Despite this, the overall findings are in alignment with WHO and ACOG guidelines, both of which recognize the effectiveness of Foley catheters and dinoprostone for cervical ripening. Our findings support flexibility in choosing between these options based on local availability, clinical expertise, and patient-specific factors. We recommend the production of further large-scale RCTs with blinded outcomes detectors to provide results with a minimized risk of detection bias. These trials are recommended to stratify the results by the women’s parity and previous caesarian delivery, dinoprostone preparation, dosage, and duration of application, and Foley’s catheter size and balloon inflation.

## 5. Conclusions

This review concludes a comparable efficacy of topical dinoprostone and transcervical Foley’s catheter in cervical ripening among women with an unripe cervix and an indication for IOL. Foley’s catheter-mediated ripening is linked to lower risks of uterine hyperstimulation, caesarian delivery, postpartum hemorrhage, and a 1-min Apgar score < 7. In addition, Foley’s catheter-mediated ripening increases the need for labor augmentation with oxytocin. Foley’s catheter is suitable for cervical ripening among women with a previous caesarian delivery and in low-resource settings. This review highlights that both topical dinoprostone and transcervical Foley catheter are effective options for cervical ripening, with comparable safety profiles. Clinicians can consider either method based on patient-specific factors, available resources, and institutional protocols. Further large-scale RCTs are required to allow for the identification of the best dose and preparation of dinoprostone, and the best Foley’s catheter size and extent of balloon inflation, as well as the best duration of each intervention application.

## Figures and Tables

**Table 1:** Summary of the included studies.

Study ID	Study Design	Country	Sample Size (Foley’s Catheter/Dinoprostone)	Eligibility Criteria	Foley’s Catheter Size (French)	Foley’s Balloon Inflation (mL)	Dinoprostone Preparation	Dinoprostone Dose (mg)
Liu et al., 2024 [27]	RCT	China	1860 (930/930)	Women with a live singleton term pregnancy in cephalic presentation with intact membranes and a Bishop score < 6 who were planned for IOL were included. Women with a previous CS, any contraindication for vaginal delivery, or nonreassuring fetal assessment were excluded.	-	60	Controlled-release insert	10
Nipanal et al., 2024 [28]	Non-randomized trial	India	182 (91/91)	Women with a live singleton term pregnancy in cephalic presentation with intact membranes, no uterine contractions, and an indication for IOL were included. Women with gestational diabetes or hypertension, oligo- or polyhydramnios, fetal distress or malpresentation, IUGR, a previous CS, or a previous uterine surgery were excluded.	18	50	Gel	1.25
Chawla et al., 2023 [29]	RCT	India	200 (100/100)	Women with a singleton pregnancy over 37 weeks of gestation in cephalic presentation with intact membranes, a Bishop score < 6, and a valid indication for IOL were included. Women with a scarred uterus, APH, chorioamnionitis, or any contraindication of vaginal delivery were excluded.	-	60	Gel	0.5
Dalal et al., 2023 [30]	RCT	India	200 (100/100)	Women with a singleton pregnancy ≥ 28 weeks of gestation in cephalic presentation with intact membranes, a Bishop score ≤ 3, and an indication for IOL were included. Women with a pre-existing medical disorder, a scarred uterus, APH, placenta previa, or contracted pelvis were excluded.	-	50	Gel	-
Kulsoom et al., 2023 [31]	Prospective cohort	India	85 (50/35)	Women with a singleton pregnancy over 34 weeks of gestation in cephalic presentation with intact membranes, a Bishop score ≤ 6, reactive non-stress test, a previous LSCS, and a valid indication for IOL were included. Women with any contraindication of prostaglandins or vaginal delivery, latex allergy, previous uterine surgery other than LSCS, FHR abnormality, active genital tract infection, or abnormal placental location were excluded.	-	60–90	Controlled-release insert	10
Poonia et al., 2023 [32]	RCT	India	60 (30/30)	Women with a singleton pregnancy ≥ 37 weeks of gestation with a Bishop score ≤ 6, a previous LSCS within 18 months, and an indication for IOL were included. Women with a contraindication of prostaglandins, previous classical uterine scar or more than one LSCS, impending eclampsia, or active lower genital tract infection were excluded.	16	30	Gel	0.5
Socha et al., 2023 [33]	Retrospective cohort	Poland	2100 (845/1255)	Women with a live singleton pregnancy > 41 weeks of gestation in cephalic presentation with a Bishop score of 3–5 and an indication for IOL were included. Women with a previous CS, PROM, or any contraindication of vaginal delivery or IOL were excluded.	-	80–100	Controlled-release insert	10
Vimarshitha et al., 2023 [34]	RCT	India	120 (60/60)	Primi- or 2nd gravida women with a singleton term pregnancy in cephalic presentation, from 37 to 40 weeks of gestation, with a Bishop score < 6, a reassuring FHR, and an indication for IOL were included. Women with previous uterine surgery, oligohydramnios, placenta previa, cord prolapse, chorioamnionitis, or herpes were excluded.	-	30–50	Gel	0.5
Mlodawski et al., 2021 [35]	Retrospective cohort	Poland	456 (356/100)	Women with a live singleton term pregnancy in cephalic presentation with intact membranes, a Bishop score < 7, and an indication for IOL were included. Women who underwent a previous CS were excluded.	20	50–60	Controlled-release insert	10
Athiel et al., 2020 [36]	Retrospective cohort	France	238 (107/131)	Women with a singleton cephalic pregnancy, a Bishop score ≤ 6, and a prelabor rupture of membranes were included. Women with any contraindication to vaginal delivery or a previous uterine surgery were excluded.	22	50	Pessary/gel	10/3
Blair et al., 2020 [37]	Retrospective cohort	Canada	153 (82/71)	Women with a live singleton pregnancy who underwent outpatient IOL were included. Women younger than 18 years and women with a previous CS or a contraindication to the interventions were excluded.	-	30–60	Controlled-release insert	10
Manly et al., 2020 [38]	Retrospective cohort	Canada	229 (95/134)	Multiparous women with a singleton pregnancy ≥ 36 weeks of gestation and an indication for IOL were included. Women with a previous CS were excluded.	16	60	Controlled-release insert/Gel	10/2 or 3
Amin et al., 2018 [39]	Prospective cohort	India	72 (8/64)	Women with an IUFD beyond 20 weeks of gestation were included. Women with multiple pregnancy were excluded.	-	-	Gel	-
Chai et al., 2018 [40]	RCT	China	160 (80/80)	Women with a Bishop score ≤ 6 and an indication for IOL were included. Women with a previous cervical surgery, medical or surgical complication, PROM, placenta previa, or vaginal infection were excluded.	-	150	Suppository	10
Villalain et al., 2018 [41]	Retrospective cohort	Spain	148 (71/77)	Women with a singleton pregnancy requiring IOL for late-onset IUGR were included. Pregnancies with chromosomal anomalies, congenital malformations, or congenital infections were excluded.	-	30	Controlled-release insert	10
Barda et al., 2017 [42]	RCT	Israel	300 (150/150)	Women with a singleton pregnancy ≥ 37 weeks of gestation in cephalic vertex presentation with intact membranes, a Bishop score < 5, and a parity 1 to 3 who were planned for IOL were included. Women with a previous CS, lack of prenatal care, and any contraindication for vaginal delivery were excluded.	22	80	Tablet	3
Mizrachi et al., 2016 [43]	Case-control	Israel	346 (173/173)	Nulliparous women with a singleton pregnancy, a Bishop score < 6, and an indication for IOL were included. Women with PROM, major fetal anomalies, or stillbirth were excluded.	22	80	Tablet	3
Mohr-sasson et al., 2016 [44]	Retrospective cohort	Israel	1162 (852/310)	Women with a live singleton pregnancy beyond 24 weeks of gestation with intact membranes, a Bishop score < 6, and an indication for IOL were included. Women with major fetal congenital anomalies, previous CS or other uterine surgery, or any contraindication for vaginal delivery were excluded.	-	-	Controlled-release insert	-
Edwards et al., 2014 [45]	RCT	The US	376 (185/191)	Women with a live singleton pregnancy ≥ 36 weeks of gestation in cephalic presentation with intact membranes and unfavorable cervix (<3 cm dilatation, if 2 cm dilated <80% effaced) who underwent IOL were included. Women younger than 18 years, with uterine contractions every 5 min or more frequently, a previous uterine surgery, fever, immune dysfunction, lethal fetal anomaly, category II or III fetal heart rate pattern, undiagnosed APH, any contraindication for vaginal delivery or the intervention agents, or a previous IOL attempt during the current pregnancy were excluded.	16	30	Controlled-release insert	10
Ghanaie et al., 2013 [46]	RCT	Iran	239 (121/118)	Primiparous women with a singleton term pregnancy in cephalic vertex presentation with intact membranes, a Bishop score ≤ 4, ≤2 painful contractions per 20 min, and an indication for IOL were included. Women with evidence of spontaneous labor, significant vaginal bleeding, FHR abnormality, IOL contraindication of vaginal delivery, or failure of successful Foley catheter placement were excluded.	22	30	Suppository	0.5
Henry et al., 2013 [47]	RCT	Australia	101 (50/51)	Women with a singleton pregnancy ≥ 37 weeks of gestation in cephalic vertex presentation with a Bishop score < 7, cervical dilatation < 2 cm, and an indication for IOL were included. Women younger than 18 years, with a need for admission, contraindication to intervention agents, or a previous IOL attempt during the current pregnancy were excluded.	16	30	Gel	1 or 2
Jozwiak et al., 2013 [48]	RCT	The Netherlands	226 (107/119)	Women over 18 years of age with term pregnancy, intact membranes, and a Bishop score < 6 requiring IOL were included. Women with a previous CS, allergy to intervention agents, or a lethal congenital anomaly were excluded.	-	30	Controlled-release insert	10
Laddad et al., 2013 [49]	RCT	India	400 (200/200)	Nulliparous women with a singleton term pregnancy in cephalic presentation, intact membranes, a Bishop score ≤ 3, and an indication for IOL were included. Women with APH or medical disorders were excluded.	-	-	Gel	-
Ziyauddin et al., 2013 [50]	Non-randomized trial	India	70 (35/35)	Women with a singleton term pregnancy in cephalic presentation, a previous CS, and a Bishop score ≤ 6 were included. Women with ruptured membranes, nonreassuring fetal testing, IUFD, polyhydramnios, placenta previa, or any contraindication for IOL were excluded.	16	30	Gel	0.5
Cromi et al., 2011 [51]	RCT	Italy	396 (264/132)	Women with a live singleton pregnancy ≥ 34 weeks of gestation in cephalic vertex presentation, intact membranes, a Bishop score ≤ 6, and reassuring fetal heart tracing were included. Women with APH, previous uterine surgery, allergy to latex, placenta previa, or any other contraindication to vaginal delivery were excluded.	18	50	Controlled-release insert	10
Deshmukh et al., 2011 [52]	RCT	India	400 (200/200)	Nulliparous women with a singleton term pregnancy in cephalic presentation, intact membranes, a Bishop score ≤ 3, and an indication for IOL were included. Women with APH or medical disorders were excluded.	-	-	Gel	-
Jozwiak et al., 2011 [53]	RCT	The Netherlands	819 (411/408)	Women over 18 years of age with a live singleton term pregnancy in cephalic presentation, intact membranes, and a Bishop score < 6 requiring IOL were included. Women with a previous CS, placenta previa, allergy to intervention agents, or a lethal congenital anomaly were excluded.	16 or 18	30	Gel	1
Prager et al., 2008 [54]	RCT	Sweden	389 (198/191)	Women with a term pregnancy, a Bishop score ≤ 6, and an indication for IOL were included. Women with spontaneous contractions, a previous uterine surgery, breech presentation, an indication for immediate delivery, or a contraindication for prostaglandins or vaginal delivery were excluded.	-	50	Gel	2
Dalui et al., 2005 [55]	RCT	India	100 (50/50)	Women with a live singleton pregnancy in cephalic presentation, between 33 and 42 weeks of gestation, intact membranes, and a Bishop score < 4 were included. Women with scarred uterus, APH, placenta previa, cervicovaginal infection, or a history of cardiac disease, glaucoma, convulsive disorder, asthma, or jaundice were excluded.	16	30	Gel	0.5
Al-Taani et al., 2004 [56]	RCT	Jordan	147 (72/75)	Grand multiparous women with a term singleton pregnancy in cephalic vertex presentation, intact membranes, reassuring fetal heart tracings, a Bishop score ≤ 5, and an indication for IOL were included. Women with a previous CS, contraindications for vaginal birth, suspected CPD, or unexplained APH were excluded.	18	50	Tablet	3
Moini et al., 2003 [57]	RCT	Iran	70 (35/35)	Women with a live singleton pregnancy in cephalic presentation between 37 and 42 weeks of gestation, intact membranes, and a Bishop score < 6 were included. Women with APH, active genital herpes infection, CPD, previous CS, a history of infertility, a previous IOL attempt in the current pregnancy, or any indication for emergency termination of pregnancy were excluded.	22	30	Gel	0.5
Niromanesh et al., 2003 [58]	RCT	Iran	89 (45/44)	Women between 20 and 30 years of age with a term singleton pregnancy ≥ 40 weeks of gestation, a Bishop score < 5, gravidity 1 to 3, and <6 contractions per hour were included. Women with APH, high blood pressure, a history of preterm labor or CS, active herpes simplex infection, acute polyhydramnios or oligohydramnios, chronic medical conditions, or a contraindication for the use of prostaglandin were excluded.	14	30	Tablet	3
Ben-aroya et al., 2002 [59]	Retrospective cohort	Israel	216 (161/55)	Women with a pregnancy between 36 and 42 weeks of gestation in cephalic vertex presentation and a previous LSCS were included. Women planned for caesarian delivery and women who received more than one modality of cervical ripening were excluded.	-	50–60	Tablet	0.5
Ghezzi et al., 2001 [60]	Non-randomized trial	Italy	224 (122/102)	Women with a singleton pregnancy beyond 37 weeks of gestation in cephalic presentation, intact membranes, a Bishop score < 4, and negative *Streptococcus agalactiae* vaginal swabs were included. Women with spontaneous uterine contractions, placenta previa, unexplained vaginal bleeding, suspected chorioamnionitis, active genital herpes, or previous uterine surgery were excluded.	18	50	Gel	0.5
Sciscione et al., 1999 [61]	RCT	The US	149 (77/72)	Women with a live singleton pregnancy beyond 28 weeks of gestation in cephalic vertex presentation, intact membranes, reactive FHR, <6 contractions per hour, and a Bishop score ≤ 5 were included. Women with APH, placenta previa, PGE2 latex allergy, or a previous IOL attempt in the current pregnancy were excluded.	14	30	Gel	0.5
Hemlin et al., 1998 [62]	RCT	Sweden	85 (43/42)	Women with ≥36 weeks’ pregnancy, a modified Bishop score ≤ 5, and an indication for IOL were included. Women with signs of cervicitis, placenta previa, 2nd or 3rd trimester vaginal bleeding, malpresentation, or fetal weight > 4500 were excluded.	24	30	-	0.5
Orhue et al., 1995 [63]	RCT	Nigeria	60 (30/30)	Nulliparous women with a live singleton term pregnancy in cephalic vertex presentation, a Bishop score ≤ 4, and an indication for IOL were included. Women with CPD, <155 cm height, >35 years of age, previous uterine surgery, anemia or known hemoglobinopathy, fetal anomalies, polyhydramnios, placenta previa or placental abruption, a contraindication of prostaglandins, or a previous IOL attempt in the current pregnancy were excluded.	17	30	Pessary	3
St. Onge et al., 1995 [64]	RCT	Canada	62 (34/28)	Women with a live singleton pregnancy between 37 and 43 weeks of gestation in cephalic vertex presentation, intact membranes, and a Bishop score ≤ 4 were included. Women with previous uterine surgery, a history of preterm labor, placenta previa, APH, or any contraindication to the use of PGE 2 gel were excluded.	18	30	Gel	0.5
Lyndrup et al., 1994 [65]	RCT	Denmark	109 (59/50)	Women with a singleton pregnancy in cephalic presentation, intact membranes, cervical length ≥ 1 cm, internal os width ≤ 1 fingertip, and an indication for IOL were included. Women with a pathological non-stress test, placenta previa, cardiopulmonary diseases, glaucoma, or previous uterine surgery were excluded	23	30	Pessary	2.5
Lewis et al., 1983 [66]	RCT	The UK	44 (22/22)	Women with a singleton pregnancy in cephalic presentation, a Bishop score ≤ 4, and an indication for IOL were included.	14	30	Pessary	3
Ezimokhai et al., 1980 [67]	Prospective cohort	The US	35 (21/14)	Primigravidae with a modified Bishop score of 0 to 3 and an indication for IOL were included.	17	35–40	Gel	5

APH: antepartum hemorrhage, CS: caesarian section, CVD: cephalopelvic disproportion, FHR: fetal heart rate, IOL: induction of labor, IUFD: intrauterine fetal death, IUGR: intrauterine growth restriction, LSCS: lower-segment caesarian section, PROM: premature rupture of membranes, UK: United Kingdom, US: United States.

**Table 2:** Baseline characteristic on the enrolled participants.

Study ID	Age (Years)		BMI (kg/m^2^)		Gestational Age (Weeks)		Primigravida		Bishop Score		Indication for Induction									
											Postmaturity		Gestational Hypertension		Gestational Diabetes		IUGR		Oligo-/Polyhydramnios	
	Mean ± SD		Mean ± SD		Mean ± SD		n (%)		Mean ± SD		n (%)		n (%)		n (%)		n (%)		n (%)	
	Foley’s Catheter	Dinoprostone	Foley’s Catheter	Dinoprostone	Foley’s Catheter	Dinoprostone	Foley’s Catheter	Dinoprostone	Foley’s Catheter	Dinoprostone	Foley’s Catheter	Dinoprostone	Foley’s Catheter	Dinoprostone	Foley’s Catheter	Dinoprostone	Foley’s Catheter	Dinoprostone	Foley’s Catheter	Dinoprostone
Liu et al., 2024 [27]	30.4 ± 3.4	30.6 ± 3.7	26.9 ± 3.2	26.8 ± 3.1	40.3 ± 1	40.3 ± 1	763 (82%)	759 (81.6%)	1:2 (0.2%), 2:15 (1.6%), 3:105 (11.3%), 4:752 (80.9%), 5:56 (6%)	1:1 (0.1%), 2:16 (1.7%), 3:97 (10.4%), 4:759 (81.6%), 5:57 (6.1%)	434 (46.7%)	448 (48.2%)	63 (6.8%)	65 (7%)	203 (21.8%)	184 (19.8%)	15 (1.6%)	14 (1.5%)	98 (10.5%)	96 (10.3%)
Nipanal et al., 2024 [28]	23.7	24.1	-	-	40	39.6	60 (65.9%)	56 (61.5%)	2.18	2.95	-	-	0 (0%)	0 (0%)	0 (0%)	0 (0%)	0 (0%)	0 (0%)	0 (0%)	0 (0%)
Chawla et al., 2023 [29]	23.8 ± 3.2	23.5 ± 3.3	-	-	39.6 ± 1.2	39.8 ± 1.2	71 (71%)	79 (79%)	2 ± 0.9	2.2 ± 1.3	-	-	-	-	-	-	-	-	-	-
Dalal et al., 2023 [30]	25.3 ± 3.9	25.7 ± 3.8	-	-	39.5 ± 1.3	39.6 ± 1.5	58 (58%)	52 (52%)	-	-	38 (38%)	45 (45%)	29 (29%)	28 (28%)	-	-	-	-	32 (32%)	26 (26%)
Kulsoom et al., 2023 [31]	28.3 ± 3.7	27.5 ± 3.2	-	-	39.3 ± 1.4	39.7 ± 1.3	0 (0%)	0 (0%)	3.4 ± 0.6	3.2 ± 0.4	12 (24%)	8 (22.9%)	10 (20%)	11 (31.4%)	-	-	6 (12%)	3 (8.6%)	5 (10%)	6 (17.1%)
Poonia et al., 2023 [32]	27.2 ± 3.2	27.4 ± 2.5	-	-	39.5 ± 1.7	39.3 ± 1.4	0 (0%)	0 (0%)	3.1 ± 0.7	3.2 ± 1.2	11 (36.7%)	7 (23.3%)	8 (26.7%)	6 (20%)	-	-	3 (10%)	2 (6.7%)	2 (6.7%)	2 (6.7%)
Socha et al., 2023 [33]	29.3 ± 12.6		-		-		1819 (63%)		-		-		-		-		-		-	
Vimarshitha et al., 2023 [34]	-	-	-	-	37–40:31 (51.7%) 40–42:29 (48.3%)	37–40:33 (55%) 40–42:27 (45%)	-	-	2.3 ± 0.7	2.3 ± 0.6	32 (53.3%)	24 (40%)	17 (26.3%)	15 (25%)	0 (0%)	0 (0%)	0 (0%)	0 (0%)	0 (0%)	0 (0%)
Mlodawski et al., 2021 [35]	28.4 ± 4.7	29 ± 3.6	-	-	40 ± 0.6	40 ± 0.5	279 (78.4%)	75 (75%)	-	-	242 (68%)	63 (63%)	46 (13%)	9 (9%)	50 (14%)	16 (16%)	-	-	-	-
Athiel et al., 2020 [36]	29.9 ± 5.1	28.3 ± 5.4	24.6 ± 4.9	24.8 ± 4.7	39.3 ± 1.1	39.3 ± 1.2	71 (66.4%)	93 (70%)	2.2 ± 1.2	2.3 ± 1.2	-	-	-	-	-	-	-	-	-	-
Blair et al., 2020 [37]	30.4 ± 5.4	30.6 ± 5.4	27.1 ± 6.1	27.1 ± 7.8	37–40:18 (21.9%) ≥40:64 (78.1%)	37–40:12 (16.9%) ≥40:59 (83.1%)	51 (62.2%)	48 (67.6%)	-	-	66 (80.5%)	63 (88.7%)	-	-	-	-	-	-	-	-
Manly et al., 2020 [38]	35 ± 4.7	34.3 ± 4.6	25.5 ± 6.1	27.2 ± 6.8	38.8 ± 1.3	39 ± 1.4	0 (0%)	0 (0%)	-	-	9 (9.5%)	24 (17.9%)	6 (6.3%)	11 (8.2%)	10 (10.5%)	21 (15.7%)	16 (16.8%)	7 (5.2%)	10 (10.5%)	2 (1.5%)
Amin et al., 2018 [39]	-	-	-	-	20.1–24:2 (3.1%) 24.1–28:44 (68.8%) 36.1–40:15 (23.4%) >40:3 (4.7%)	20.1–24:0 (0%) 24.1–28:6 (75%) 36.1–40:2 (25%) >40:0 (0%)	47 (47%)		-	-	0 (0%)	0 (0%)	0 (0%)	0 (0%)	0 (0%)	0 (0%)	0 (0%)	0 (0%)	0 (0%)	0 (0%)
Chai et al., 2018 [40]	24.9 ± 4.2	25.3 ± 4.4	-	-	38.9 ± 2.6	38.5 ± 2.3	-	-	3.3 ± 0.4	3.4 ± 0.5	6 (7.5%)	9 (11.3%)	-	-	-	-	-	-	33 (41.3%)	30 (37.5%)
Villalain et al., 2018 [41]	32.1 ± 6.2	32.5 ± 6.3	25.2 ± 5.3	24.3 ± 5	37.9 ± 1.1	37.6 ± 1.4	50 (70.4%)	60 (77.9%)	3 ± 3	4 ± 1.5	0 (0%)	0 (0%)	0 (0%)	0 (0%)	0 (0%)	0 (0%)	100 (100%)	100 (100%)	0 (0%)	0 (0%)
Barda et al., 2017 [42]	-	-	-	-	-	-	-	-	-	-	60 (40%)	73 (48.7%)	-	-	-	-	-	-	-	-
Mizrachi et al., 2016 [43]	26.9 ± 4.4	27.6 ± 5.4	20.7 ± 9.3	21.6 ± 9	40.3 ± 1.3	40.2 ± 1.4	173 (100%)	173 (100%)	-	-	136 (39.3%)		36 (10.4%)		10 (2.9%)		38 (11%)		-	
Mohr-sasson et al., 2016 [44]	31.3 ± 5.2	31.7 ± 6	28.2 ± 4.2	29.3 ± 5.1	39 ± 1.5	38.7 ± 1.5	-	-	-	-	-	-	-	-	-	-	-	-	-	-
Edwards et al., 2014 [45]	28 ± 6.4	26.9 ± 5.9	35.6 ± 8.6	36.1 ± 9.3	39.1 ± 1.4	39.2 ± 1.5	106 (57%)	127 (66%)	-	-	-	-	-	-	-	-	-	-	-	-
Ghanaie et al., 2013 [46]	24.1 ± 2	22.5 ± 4	-	-	39.1 ± 1.4	38.9 ± 1.9	121 (100%)	118(100%)	3.1 ± 1.9	3.3 ± 1.5	19 (15.7%)	20 (16.9%)	17 (14%)	19 (16.1%)	3 (2.5%)	5 (4.2%)	9 (7.4%)	11 (9.3%)	21 (17.4%)	17 (14.4%)
Henry et al., 2013 [47]	32.7	32.9	24.1	23	40.8	40.6	45 (90%)	46 (90%)	2.7 ± 1.7	2.9 ± 1.7	38 (76%)	35 (69%)	3 (6%)	2 (4%)	3 (6%)	6 (12%)	-	-	-	-
Jozwiak et al., 2013 [48]	30.5 ± 4	31.7 ± 5.2	25.3 ± 4.6	24.5 ± 4.4	39.3 ± 2	39.8 ± 2.1	77 (72%)	83 (70%)	0:18 (17%), 1:33 (31%), 2:27 (25%), 3:17 (16%), 4:9 (8%), 5:3 (3%)	0:19 (16%), 1:28 (24%), 2:40 (34%), 3:19 (16%), 4:8 (7%), 5:5 (4%)	20 (19%)	28 (24%)	51 (48%)	34 (29%)	7 (7%)	7 (6%)	8 (8%)	7 (6%)	7 (7%)	11 (9%)
Laddad et al., 2013 [49]	21.3 ± 3	21 ± 2.8	-	-	38.7 ± 1.7	38.9 ± 1.7	200 (100%)	200 (100%)	1.5 ± 0.7	1.6 ± 0.8	60 (30%)	57 (28.5%)	78 (39%)	74 (37%)	-	-	12 (6%)	14 (7%)	10 (5%)	5 (2.5%)
Ziyauddin et al., 2013 [50]	28.4 ± 15.5	28.7 ± 15.5	-	-	39.4 ± 3.9	39.3 ± 3.9	0 (0%)	0 (0%)	2.8	3	8 (22.9%)	7 (20%)	9 (25.7%)	11 (31.4%)	-	-	9 (25.7%)	5 (14.3%)	-	-
Cromi et al., 2011 [51]	32 ± 4.7	31 ± 4.9	28.7 ± 5	28.3 ± 4.7	39.8 ± 1.8	39.8 ± 2	183 (69.3%)	89 (67.4%)	2 ± 0.9	2 ± 0.8	79 (29.9%)	43 (32.6%)	35 (13.2%)	12 (9%)	-	-	17 (6.4%)	11 (8.3%)	51 (19.3%)	32 (24.2%)
Deshmukh et al., 2011 [52]	22.3 ± 3	22 ± 3	-	-	38.7 ± 1.7	38.9 ± 1.7	200 (100%)	200 (100%)	1.5 ± 0.7	1.6 ± 0.8	59 (29.5%)	62 (31%)	74 (37%)	73 (36.5%)	-	-	10 (5%)	11 (5.5%)	10 (5%)	1 (0.5%)
Jozwiak et al., 2011 [53]	30·9 ± 4·9	30·6 ± 5	25.3 ± 1.2	24.8 ± 1.3	40.1 ± 0.5	40 ± 0.5	268 (65%)	263 (65%)	0:38 (9%), 1:103 (25%), 2:115 (28%), 3:91 (22%), 4:53 (13%), 5:11 (3%)	0:52 (13%), 1:85 (21%), 2:112 (28%), 3:83 (20%), 4:56 (14%), 5:20 (5%)	147 (36%)	143 (35%)	140 (34%)	140 (34%)	24 (6%)	34 (8%)	33 (8%)	17 (4%)	32 (8%)	27 (7%)
Prager et al., 2008 [54]	32.4	33.3	-	-	40.3	40.2	120 (61%)	131 (69%)	3.4	3.2	76 (38%)	65 (34%)	-	-	-	-	-	-	-	-
Dalui et al., 2005 [55]	-	-	-	-	33–36:12 (24%) 37–40:38 (76%)	33–36:11 (22%) 37–40:39 (78%)	32 (64%)	39 (78%)	2.4 ± 1	2.7 ± 0.9	3 (6%)	4 (8%)	22 (44%)	23 (46%)	1 (2%)	5 (10%)	11 (22%)	8 (16%)	-	-
Al-Taani et al., 2004 [56]	27.7 ± 5.5	27.1 ± 5.7	-	-	39.4 ± 1.9	39.5 ± 1.7	0 (0%)	0 (0%)	2.6 ± 1.4	2.6 ± 1.3	29 (40%)	23 (31%)	20 (28%)	24 (32%)	8 (11%)	12 (16%)	10 (14%)	11 (15%)	-	-
Moini et al., 2003 [57]	22.4 ± 3.2	23.1 ± 3.2	-	-	40.7 ± 0.5	40.6 ± 0.5	35 (100%)	35 (100%)	3.9 ± 0.8	3.3 ± 0.7	30 (85.7%)	32 (91.4%)	2 (5.7%)	1 (2.9%)	-	-	1 (2.9%)	1 (2.9%)	2 (5.7%)	1 (2.9%)
Niromanesh et al., 2003 [58]	23.9 ± 3.5	24 ± 3.5	-	-	40.8 ± 0.4	40.8 ± 0.4	-	-	3.2 ± 1.1	3.2 ± 1.2	-	-	-	-	-	-	-	-	-	-
Ben-aroya et al., 2002 [59]	28.1 ± 4.9	29.3 ± 5.1	-	-	39.8 ± 1.2	39.2 ± 1.4	0 (0%)	0 (0%)	-	-	-	-	-	-	-	-	-	-	-	-
Ghezzi et al., 2001 [60]	28.7 ± 6	29.1 ± 5.2	-	-	39.8 ± 2.3	39.6 ± 2.1	84 (68.9%)	62 (60.8%)	2.2 ± 1.1	2.3 ± 1.1	48 (39.3%)	44 (43.1%)	28 (23%)	20 (19.6%)	3 (2.5%)	2 (2%)	21 (17.2%)	15 (14.2%)	12 (9.8%)	12 (11.8%)
Sciscione et al., 1999 [61]	26.3 ± 48.2	26.3 ± 55.2	-	-	38.3 ± 22.8	38.4 ± 39	-	-	2.8 ± 14.9	2.4 ± 11	-	-	-	-	-	-	-	-	-	-
Hemlin et al., 1998 [62]	29.3 ± 5.3	28.4 ± 5.8	-	-	39.4 ± 1.8	39.3 ± 1.5	20 (46.5%)	21 (50%)	2.4 ± 1 ^	2.4 ± 1 ^	12 (27.9%)	11 (26.2%)	19 (44.2%)	16 (38.1%)	1 (2.3%)	2 (4.8%)	5 (11.6%)	4 (9.5%)	-	-
Orhue et al., 1995 [63]	21 ± 4.5	22 ± 5	-	-	41.3 ± 2.2	41.22 ± 2.1	30 (100%)	30 (100%)	2.1 ± 0.8	2 ± 0.8	18 (60%)	16 (53.3%)	12 (40%)	10 (33.3%)	0 (0%)	0 (0%)	2 (6.7%)	2 (6.7%)	0 (0%)	0 (0%)
St. Onge et al., 1995 [64]	27.9 ± 5.8	28.9 ± 5.8	-	-	39.7 ± 1.7	39.8 ± 1.6	28 (82.3%)	21 (75%)	2.9 ± 1.7	2.5 ± 1.6	2 (5.9%)	3 (10.7%)	16 (47%)	8 (28.6%)	3 (8.8%)	3 (10.7%)	2 (5.9%)	2 (7.1%)	11 (32.3%)	12 (42.9%)
Lyndrup et al., 1994 [65]	27.1 ± 5.8	26.7 ± 3.5	-	-	40.1 ± 2	40 ± 1.8	44 (74.6%)	44 (88%)	4.5 ± 1.5	4.1 ± 1.3	20 (33.9%)	15 (30%)	13 (22%)	21 (42%)	-	-	-	-	-	-
Lewis et al., 1983 [66]	25.4	-	-		40.5		17 (77.3%)	17 (77.3%)	2.6 ^		34 (51.5%)		24 (36.4%)		0 (0%)		6 (0.9%)		0 (0%)	
Ezimokhai et al., 1980 [67]	22.9 ± 4.1	23.5 ± 5.6	-	-	40.6 ± 1.1	40 ± 1.9	21 (100%)	14 (100%)	2 ± 0.8 ^	1.9 ± 0.9 ^	8 (38.1%)	5 (35.7%)	18 (85.7%)	8 (57.1%)	0 (0%)	0 (0%)	2 (9.5%)	0 (0%)	0 (0%)	0 (0%)

BMI: body mass index, IUGR: intrauterine growth restriction, SD: standard deviation. ^ modified Bishop score.

## Data Availability

Not applicable.

## Supplementary Materials

The following supporting information can be downloaded at: https://www.mdpi.com/article/10.3390/healthcare13090983/s1, Figure S1: Risk of bias graph; Figure S2: Risk of bias summary; Figure S3: Forest plot of the analysis; Instrumental vaginal delivery; Figure S4: Forest plot of the analysis; Time from induction to birth (hours); Figure S5: Forest plot of the analysis; Vaginal delivery within 24 h; Figure S6: Forest plot of the analysis; Induction failure; Figure S7: Forest plot of the analysis; Bishop score change; Figure S8: Forest plot of the analysis; Intrapartum pyrexia; Figure S9: Forest plot of the analysis; Postpartum infection; Figure S10: Forest plot of the analysis; Meconium passage; Figure S11: Forest plot of the analysis; Umbilical cord arterial pH 7.1; Figure S12: Forest plot of the analysis; NICU admission. Table S1: Quality assessment of cohort studies; Table S2: Quality assessment of case-control studies.
