# Supplementary material for: Topical Dinoprostone vs. Foley’s Catheter: A Systematic Review and Meta-Analysis of Cervical Ripening Approaches
Source: Healthcare (Basel). 2025 Apr 24;13(9):983. doi: 10.3390/healthcare13090983 (PMC12071297; doi:10.3390/healthcare13090983)
Supplement: Supplementary file 1 [file healthcare-13-00983-s001.zip › Table S1.pdf]

Table. S1: NIH Quality Assessment Tool for Observational Cohort and Cross-Sectional Studies.

Study ID	1. Was the research question or objective in this paper clearly stated?	2. Was the study population clearly specified and defined?	3. Was the participation rate of eligible persons at least 50%?	4. Were all the subjects selected or recruited from the same or similar populations (including the same time period)? Were inclusion and exclusion criteria for being in the study prespecified and applied uniformly to all participants?	5. Was a sample size justification, power description, or variance and effect estimates provided?	6. For the analyses in this paper, were the exposure(s) of interest measured prior to the outcome(s) being measured?	7. Was the time frame sufficient so that one could reasonably expect to see an association between exposure and outcome if it existed?	8. For exposures that can vary in amount or level, did the study examine different levels of the exposure as related to the outcome (eg, categories of exposure, or exposure measured as continuous variable)?	9. Were the exposure measures (independent variables) clearly defined, valid, reliable, and implemented consistently across all study participants?	10. Was the exposure(s) assessed more than once over time?	11. Were the outcome measures prespecified, clearly defined, valid, reliable, and assessed consistently across all study participants?	12. Were the people assessing the outcomes blinded to the participants' exposures/ interventions?	13. Was the loss to follow-up after baseline 20% or less? Were those lost to follow-up accounted for in the analysis?	14. Were key potential confounding variables measured and adjusted statistically for their impact on the relationship between exposure(s) and outcome(s)?	Total score	Quality rating
**Kulsoom et al. 2023 [1]**	Yes	Yes	Yes	Yes	No	Yes	Yes	No	Yes	No	Yes	No	Yes	No	11.5	**Good**
**Socha et al. 2023 [2]**	Yes	Yes	Yes	Yes	No	Yes	Yes	No	Yes	No	Yes	No	Yes	No	11.5	**Good**
**Mlodawski et al. 2021 [3]**	Yes	Yes	Yes	No	No	Yes	Yes	No	Yes	No	Yes	No	Yes	No	11	**Good**
**Athiel et al. 2020 [4]**	Yes	Yes	Yes	No	No	Yes	Yes	No	Yes	Yes	Yes	No	Yes	No	11.5	**Good**
**Blair et al. 2020 [5]**	Yes	Yes	Yes	Yes	No	Yes	Yes	No	Yes	Yes	Yes	No	Yes	Yes	12.5	**Good**

**Manly et al. 2020 [6]**	Yes	Yes	Yes	Yes	No	Yes	Yes	No	Yes	Yes	Yes	No	Yes	Yes	12.5	**Good**
**Amin et al. 2018 [7]**	Yes	Yes	Yes	Yes	No	Yes	Yes	Yes	Yes	Yes	Yes	No	Yes	No	12.5	**Good**
**Villalain et al. 2018 [8]**	Yes	Yes	Yes	No	No	Yes	Yes	No	Yes	Yes	Yes	No	Yes	Yes	12	**Good**
**Mohr-sasson et al. 2016 [9]**	Yes	Yes	Yes	Yes	No	Yes	Yes	No	Yes	No	Yes	No	Yes	Yes	12	**Good**
**Ben-aroya et al. 2002 [10]**	Yes	Yes	Yes	Yes	No	Yes	Yes	No	Yes	No	Yes	No	Yes	No	11.5	**Good**
**Ezimokhai et al. 1980 [11]**	Yes	Yes	Yes	Yes	No	Yes	Yes	No	Yes	No	Yes	No	Yes	No	11.5	**Good**

Each question is answered: Yes=1, No=0.5, Not Reported (NR), Cannot Determine (CD) or Not Applicable (NA)=0. Quality rating: good (11-14 points) or fair (7.5-10.5 points) or poor (0-7 points). NIH: national institute of health.

References

1. Kulsoom U, Parveen S, Noor N, Firdaus U. Dinoprostone Vaginal Insert vs Intracervical Foley Catheter for Preinduction Cervical Ripening in Women with Previous Cesarean Section. Journal of South Asian Federation of Obstetrics and Gynaecology. 2023;14:672-5. 10.5005/jp-journals-10006-2153

2. Socha MW, Flis W, Pietrus M, Wartęga M. Risk of Cesarean Delivery after Vaginal Inserts with Prostaglandin Analogs and Single-Balloon Catheter Used for Cervical Ripening and Induction of Labor. Biomedicines. 2023;11(8). 10.3390/biomedicines11082125

3. Mlodawski J, Mlodawska M, Armanska J. Dinoprostone vaginal insert vs the Foley catheter in labor induction. Observational study. Clinical and Experimental Obstetrics & Gynecology. 2021;48:665-9. 10.31083/j.ceog.2021.03.2498

4. Athiel Y, Crequit S, Bongiorno M, Sanyan S, Renevier B. Term prelabor rupture of membranes: Foley catheter versus dinoprostone as ripening agent. Journal of gynecology obstetrics and human reproduction. 2020;49(8):101834. 10.1016/j.jogoh.2020.101834

5. Blair R, Harvey MA, Pudwell J, Bougie O. Retrospective Comparison of PGE(2) Vaginal Insert and Foley Catheter for Outpatient Cervical Ripening. Journal of obstetrics and gynaecology Canada : JOGC = Journal d'obstetrique et gynecologie du Canada : JOGC. 2020;42(9):1103-10. 10.1016/j.jogc.2020.02.112

6. Manly E, Hiersch L, Moloney A, Berndl A, Mei-Dan E, Zaltz A, et al. Comparing Foley Catheter to Prostaglandins for Cervical Ripening in Multiparous Women. Journal of obstetrics and gynaecology Canada : JOGC = Journal d'obstetrique et gynecologie du Canada : JOGC. 2020;42(7):853-60. 10.1016/j.jogc.2019.11.001

7. Amin KV, Chauhan AR, Goel A. Current Practices of Cervical Ripening and Induction of Labour in Intrauterine Foetal Demise: An Observational Study. Journal of obstetrics and gynaecology of India. 2019;69(1):37-42. 10.1007/s13224-017-1085-1

8. Villalain C, Herraiz I, Quezada MS, Gómez Arriaga P, Simón E, Gómez-Montes E, et al. Labor Induction in Late-Onset Fetal Growth Restriction: Foley Balloon versus Vaginal Dinoprostone. Fetal diagnosis and therapy. 2019;46(1):67-74. 10.1159/000491784

9. Mohr-Sasson A, Schiff E, Sindel O, Suday RR, Kalter-Farber A, Mashiach R, et al. Second dose of PGE(2) vaginal insert versus Foley transcervical balloon for induction of labor after failure of cervical ripening with PGE(2) vaginal insert. The journal of maternal-fetal & neonatal medicine : the official journal of the European Association of Perinatal Medicine, the Federation of Asia and Oceania Perinatal Societies, the International Society of Perinatal Obstet. 2017;30(17):2074-7. 10.1080/14767058.2016.1236252

10. Ben-Aroya Z, Hallak M, Segal D, Friger M, Katz M, Mazor M. Ripening of the uterine cervix in a post-cesarean parturient: prostaglandin E2 versus Foley catheter. The journal of maternal-fetal & neonatal medicine : the official journal of the European Association of Perinatal Medicine, the Federation of Asia and Oceania Perinatal Societies, the International Society of Perinatal Obstet. 2002;12(1):42-5. 10.1080/jmf.12.1.42.45

11. EZIMOKHAI M, NWABINE JN. THE USE OF FOLEY'S CATHETER IN RIPENING THE UNFAVOURABLE CERVIX PRIOR TO INDUCTION OF LABOUR. British Journal of Obstetrics and Gynaecology. 1980;87:281-6.
