# Supplementary material for: Topical Dinoprostone vs. Foley’s Catheter: A Systematic Review and Meta-Analysis of Cervical Ripening Approaches
Source: Healthcare (Basel). 2025 Apr 24;13(9):983. doi: 10.3390/healthcare13090983 (PMC12071297; doi:10.3390/healthcare13090983)
Supplement: Supplementary file 1 [file healthcare-13-00983-s001.zip › Table S2.pdf]

**Table S2: NIH Quality Assessment Tool for Observational Case-control Studies.**

| Study ID | 1. Was the research question or objective in this paper clearly stated and appropriate? | 2. Was the study population clearly specified and defined? | 3. Did the authors include a sample size justification? | 4. Were controls selected or recruited from the same or similar population that gave rise to the cases (including the same timeframe)? | 5. Were the definitions, inclusion and exclusion criteria, algorithms or processes used to identify or select cases and controls valid, reliable, and implemented consistently across all study | 6. Were the cases clearly defined and differentiated from controls? | 7. If less than 100 percent of eligible cases and/or controls were selected for the study, were the cases and/or controls randomly selected from | 8. Was there use of concurrent controls? | 9. Were the investigators able to confirm that the exposure/risk occurred prior to the development of the condition or event that defined a participant as a case? | 10. Were the measures of exposure/risk clearly defined, valid, reliable, and implemented consistently (including the same time period) across all study participants? | 11. Were the assessors of exposure/risk blinded to the case or control status of participants? | 12. Were key potential confounding variables measured and adjusted statistically in the analyses? If matching was used, did the investigators account for matching during study analysis? | Total score | Quality rating |
|---|---|---|---|---|---|---|---|---|---|---|---|---|---|---|

| | | | | | | | | | | | | | | |
|---|---|---|---|---|---|---|---|---|---|---|---|---|---|---|
| | | | | | **participant s?** | | **those eligible?** | | | | | | | |
| **Mizrachi et al. 2016 [1]** | Yes | Yes | Yes | Yes | Yes | Yes | No | No | Yes | Yes | No | Yes | 10.5 | **Good** |

*Each question is answered: Yes=1, No=0.5, Not Reported (NR), Cannot Determine (CD) or Not Applicable (NA)=0. Quality rating: good (9.5-12 points) or fair (6.5-9 points) or poor (6-0 points). NIH: national institute of health.*

**References**

1. Mizrachi Y, Levy M, Bar J, Kovo M. Induction of labor in nulliparous women with unfavorable cervix: a comparison of Foley catheter and vaginal prostaglandin E2. Archives of gynecology and obstetrics. 2016;294(4):725-30. 10.1007/s00404-016-4026-9
