# Supplementary figures and images for: Topical Dinoprostone vs. Foley’s Catheter: A Systematic Review and Meta-Analysis of Cervical Ripening Approaches
Source: Healthcare (Basel). 2025 Apr 24;13(9):983. doi: 10.3390/healthcare13090983 (PMC12071297; doi:10.3390/healthcare13090983)

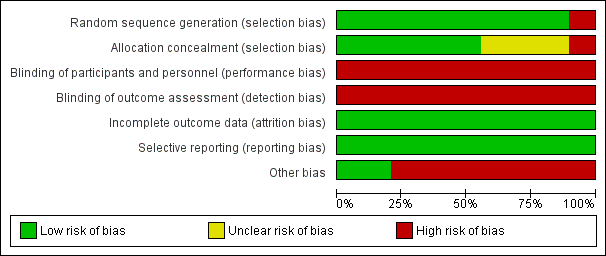

Supplement: Supplementary file 1 [file healthcare-13-00983-s001.zip › Figure S1. Risk of bias graph.png]

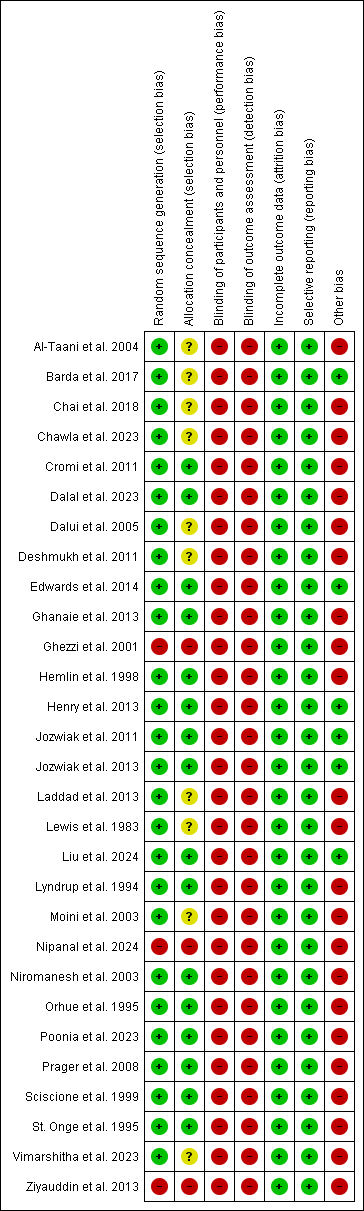

Supplement: Supplementary file 1 [file healthcare-13-00983-s001.zip › Figure S2. Risk of bias summary.png]

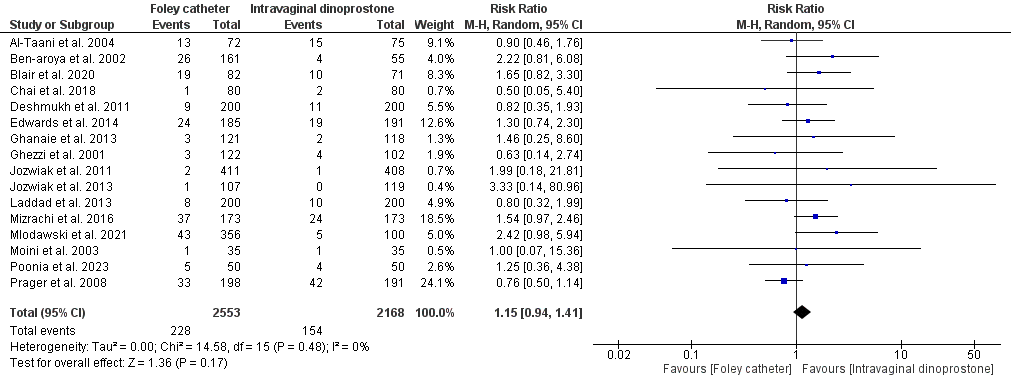

Supplement: Supplementary file 1 [file healthcare-13-00983-s001.zip › Figure. S10 Meconium passage.png]

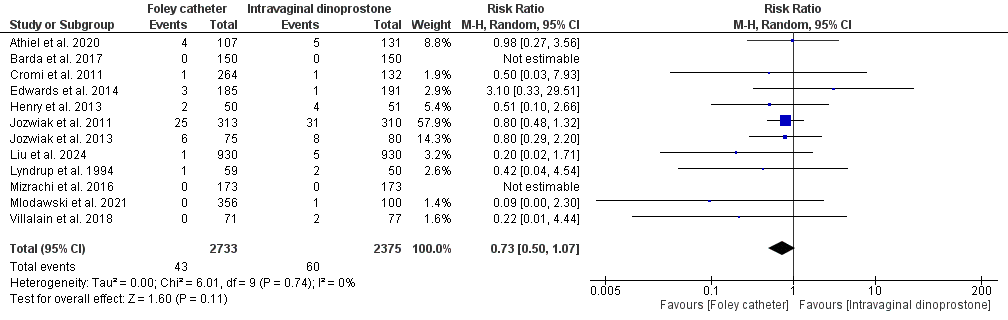

Supplement: Supplementary file 1 [file healthcare-13-00983-s001.zip › Figure. S11 Umbilical cord arterial pH 7.1 .png]

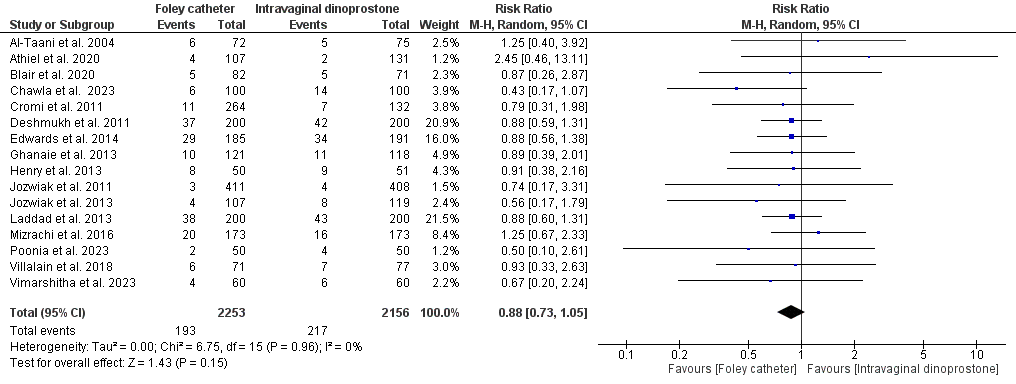

Supplement: Supplementary file 1 [file healthcare-13-00983-s001.zip › Figure. S12 NICU admission.png]

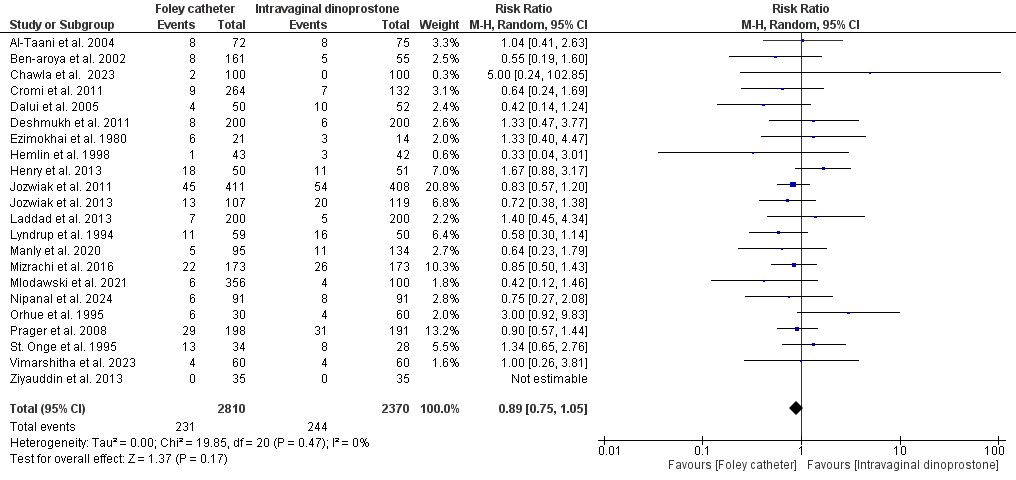

Supplement: Supplementary file 1 [file healthcare-13-00983-s001.zip › Figure. S3 Instrumental vaginal delivery.png]

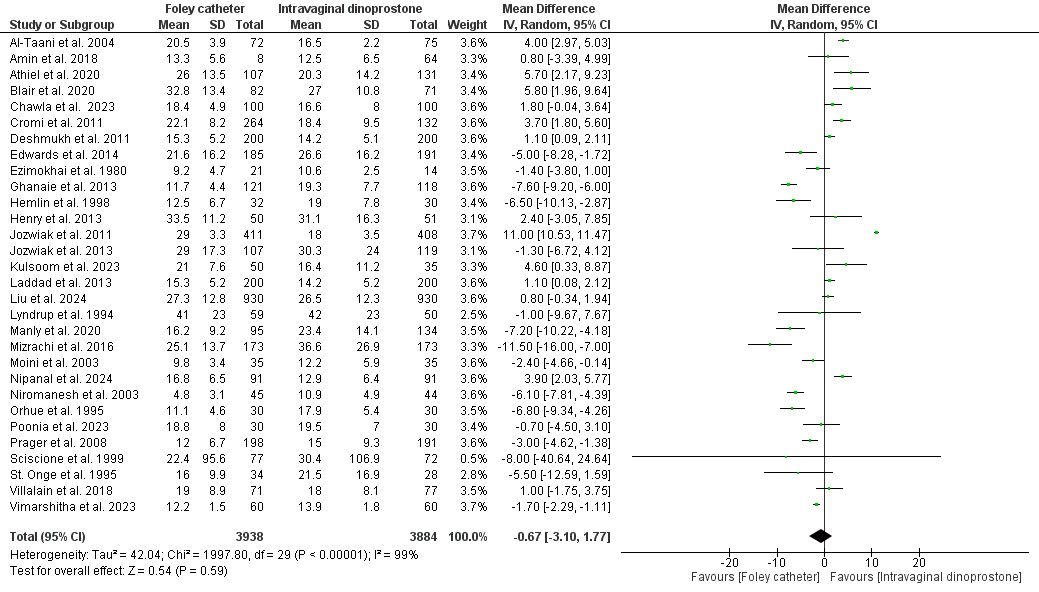

Supplement: Supplementary file 1 [file healthcare-13-00983-s001.zip › Figure. S4 Time from induction to birth (hours) .png]

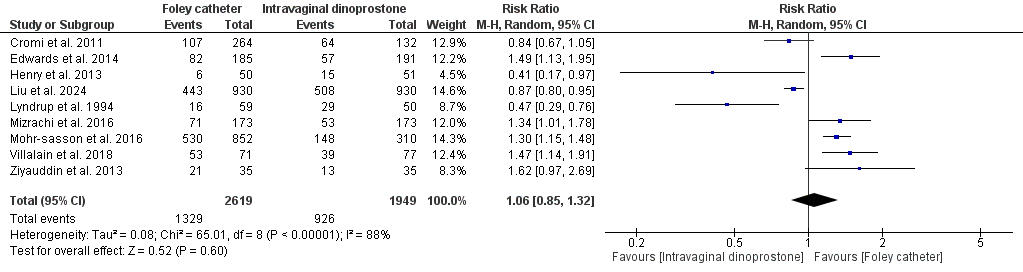

Supplement: Supplementary file 1 [file healthcare-13-00983-s001.zip › Figure. S5 Vaginal delivery within 24 hours.png]

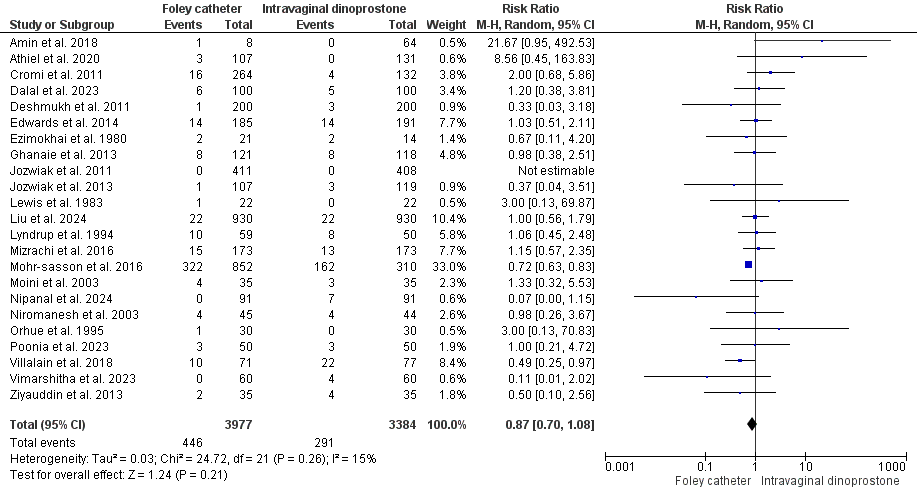

Supplement: Supplementary file 1 [file healthcare-13-00983-s001.zip › Figure. S6 Induction failure.png]

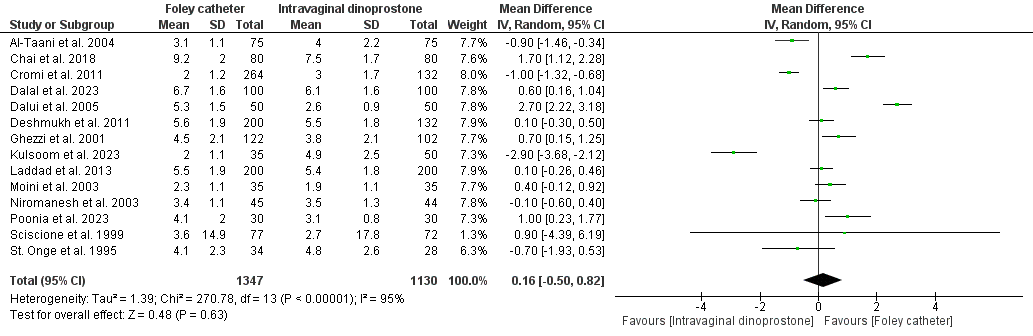

Supplement: Supplementary file 1 [file healthcare-13-00983-s001.zip › Figure. S7 Bishop score change.png]

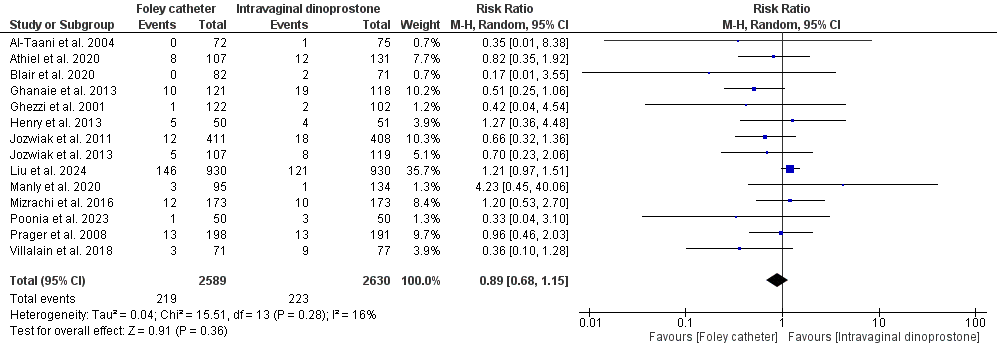

Supplement: Supplementary file 1 [file healthcare-13-00983-s001.zip › Figure. S8 intrapartum pyrexia .png]

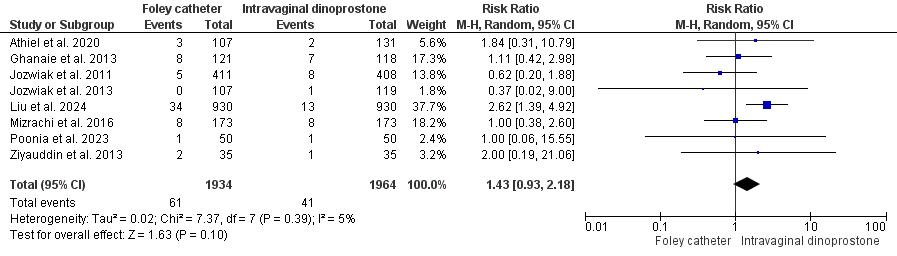

Supplement: Supplementary file 1 [file healthcare-13-00983-s001.zip › Figure. S9 Postpartum infection.png]
